# Neighbor-based adaptive sparsity orthogonal least square for fluorescence molecular tomography

**DOI:** 10.1117/1.JBO.28.6.066005

**Published:** 2023-06-29

**Authors:** Huangjian Yi, Sihao Ma, Ruigang Yang, Sheng Zhong, Hongbo Guo, Xuelei He, Xiaowei He, Yuqing Hou

**Affiliations:** a Northwest University, School of Information Sciences and Technology, Xi’an, China; b The Xi’an Key Laboratory of Radiomics and Intelligent Perception, Xi’an, China

**Keywords:** fluorescence molecular tomography, image reconstruction, neighbor strategy, orthogonal least square, support sets

## Abstract

**Significance:**

Fluorescence molecular tomography (FMT) is a promising imaging modality, which has played a key role in disease progression and treatment response. However, the quality of FMT reconstruction is limited by the strong scattering and inadequate surface measurements, which makes it a highly ill-posed problem. Improving the quality of FMT reconstruction is crucial to meet the actual clinical application requirements.

**Aim:**

We propose an algorithm, neighbor-based adaptive sparsity orthogonal least square (NASOLS), to improve the quality of FMT reconstruction.

**Approach:**

The proposed NASOLS does not require sparsity prior information and is designed to efficiently establish a support set using a neighbor expansion strategy based on the orthogonal least squares algorithm. The performance of the algorithm was tested through numerical simulations, physical phantom experiments, and small animal experiments.

**Results:**

The results of the experiments demonstrated that the NASOLS significantly improves the reconstruction of images according to indicators, especially for double-target reconstruction.

**Conclusion:**

NASOLS can recover the fluorescence target with a good location error according to simulation experiments, phantom experiments and small mice experiments. This method is suitable for sparsity target reconstruction, and it would be applied to early detection of tumors.

## 1 Introduction

Fluorescence molecular tomography (FMT) is a promising imaging technology that noninvasively and dynamically offers a 3D visualization of the biological process *in-vivo* at the cellular and molecular levels.^1^^–^^4^ Consequently, it greatly promotes its application in small animal research and preclinical diagnosis.^4^^,^^5^ However, the reconstruction of FMT is severe ill-posed caused by the strong scattering of near-infrared photons propagation in biological tissues. In addition, the number of measurements available is typically much smaller than the number of unknowns, which aggravate the under-determination of the reconstruction.^6^^,^^7^

To alleviate the ill-posedness, great effort has been made on the reconstruction algorithms. Effective regularization methods are developed, such as Tikhonov regularization, $L_{p}$-norm (0<p≤1) regularization, total variation regularization, and hybrid regularization.^8^^–^^12^ FMT is widely used in the early tumor detection. The tumor is small and sparse compared to the entire imaging domain. Tikhonov regularization, however, often provides an over-smoothed result and is absence of details in the local features.^13^^,^^14^ As a consequence, sparsity regularization methods are beneficial in this case. $L_{0}$-norm is an ideal sparsity regularizer, which can provide the sparsest solution. In this work, $L_{0}$-norm regularization has been utilized to establish the mathematical model of the inverse problem and a greedy algorithm is developed to solve this model. Greedy algorithm is a signal recovery technology for an underdetermined linear system following the principle that the sparsity can be exploited to recover signals from few samples if the system matrix satisfies the restricted isometry property.^15^^,^^16^ For example, the previously proposed orthogonal matching pursuit (OMP) is simple and effective. It selects the atom which is the most related to the current residual at each iteration.^17^ However, an inaccurate result will be obtained when the columns in the system matrix have strong correlation. Compressive sampling matching pursuit (CoSaMP) is designed for better atom selection and adopts the backtracking strategy to improve the accuracy.^18^ These algorithms need to know the sparsity in advance. However, the sparsity is usually unknown in the reconstruction process. Thong et al. proposed the sparsity adaptive matching pursuit algorithm to reconstruct the signal without prior information of the sparsity.^19^^,^^20^ This algorithm has provided the sparsity by increasing the size of the support set with a fixed step size. But it will generate an inaccurate sparsity, which further leads to incorrect results.^20^ There are substantial ongoing researches related to the field of adaptive sparse method. These studies have been applied to FMT reconstruction and achieved great success.^21^^–^^23^ In addition, the greedy algorithms mentioned above more easily get to a local optimal solution instead of the global optimal solution. In general, these methods cannot do very well in multi-target reconstruction.^17^

In practical applications, the real sparsity cannot be known in advance. Orthogonal least square (OLS) algorithm needs to input the sparsity manually based on experience.^24^ In each iteration, only one column index with the highest correlation is selected to be incorporated into the support set, which result in a worse reconstruction accuracy. In this work, neighbor-based adaptive sparsity OLS (NASOLS) is developed. This algorithm can provide the sparsity adaptively without knowing sparsity beforehand. A neighbor expansion strategy based on tetrahedron element is presented to provide the support set, which can preserve the local spatial structure information. This effective scheme has improved the resolution of double-target reconstruction. To verify the performance of NASOLS algorithm, numerical simulation experiments, phantom experiment, and small animal experiments were designed in this study. Adaptive sparsity OLS (ASOLS) without neighbor strategy, OLS, CoSaMP, and generalized OMP (gOMP) are selected as the comparative methods. The experimental results show that the NASOLS algorithm has the potential for reconstruction of FMT, especially for multi-target reconstruction.

The outline of this paper is listed as follows. The mathematical model of reconstruction problem and adaptive neighbor OLS algorithm are described in Sec. 2; Sec. 3 presents the results of simulation experiments, phantom experiments, and small animal experiments with the OLS and comparative methods; in Sec. 4, the discussions of the performance of NASOLS and conclusions of this work are shown.

## 2 Methods

### 2.1 Mathematical Model of Reconstruction Problem

In general, the light propagation is described by the radiative transfer equation.^25^ However, it is difficult to solve this complex integro-differential equation. Diffusion equation (DE) is a popular approximation in highly scattering biological tissues.^26^^,^^27^ For continuous wave FMT model, two coupled diffusion equations should be used to describe the behaviors of the excited and the emitted light. And finite element method (FEM) is utilized to solve the coupled DEs.^28^ Finally, a linear relationship between the surface photon intensity of the emitted light and the distribution of fluorescent yield inside the object can be constructed as follows:

$$\Phi^{m}=AX,$$ (1)

where $\Phi^{m}$ is the surface photon intensity, which is measured by detectors. A is the system matrix. X denotes the distribution of fluorescent yield to be reconstructed. More detailed descriptions can be found in Refs. 12, 13, and 29. Solving Eq. (1) suffers from being ill-posed. The $L_{0}$ regularization is utilized, and Eq. (1) is rewritten as follows based on compressed sensing theory:^30^

$$\underset{x}{\min}\;{\left\|X\right\|}_{0},\;\text{subject to }{\left\|AX-\Phi^{m}\right\|}_{2}^{2}<\varepsilon ,$$ (2)

where ${\left\|X\right\|}_{0}$ is the $L_{0}$-norm, and ε is a given threshold.

### 2.2 Neighbor-Based Adaptive Orthogonal Least-Squares Method

**Fig. 1:**
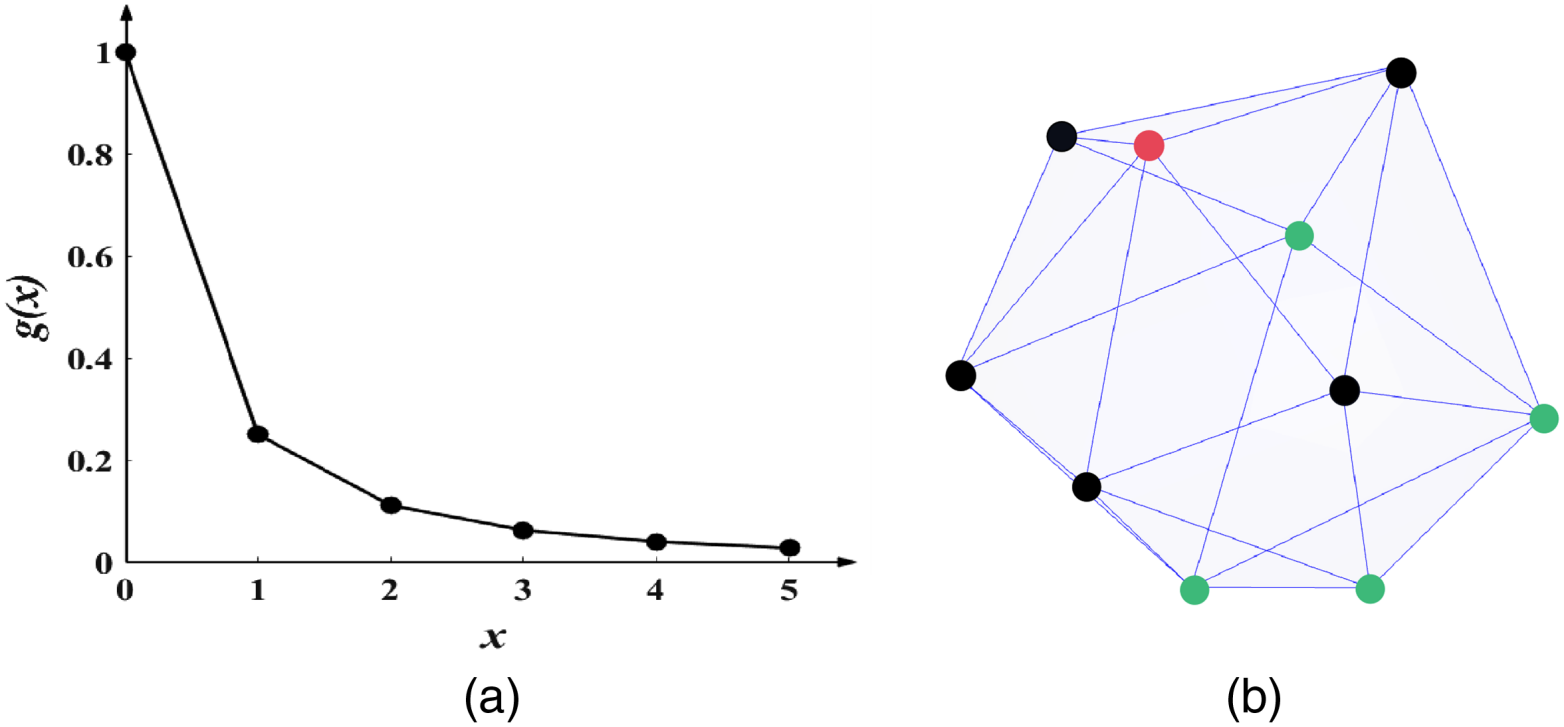
(a) Schematic diagram of exponential function. (b) Neighbors of red node are black nodes, the green nodes are the irrelevant nodes of the red node.

In this section, we present a neighbor-based adaptive OLS (NAOLS) to solve Eq. (2). The specific process of NAOLS is as follows:

1. Define column index selection formula: the critical point of this method is to select the most appropriate column indexes to add into the support set. This method sequentially projects columns of A onto a residual vector. Specifically, in the i’th iteration, the proposed method chooses new column indexes according to the equation
  $$J=\arg\;\max\;{\left\|q_{j}\right\|}_{2},$$ (3)
  where
  $$\begin{array}{c} q_{j}=\frac{a_{j}^{T}r^{i}}{a_{j}^{T}t_{j}^{i}}t_{j}^{i} \end{array},$$ (4)
  $$\begin{array}{c} t_{j}^{i+1}≜a_{j}-\overset{i}{\underset{l=1}{\sum }}\frac{a_{j}^{T}u_{l}}{{\left\|u_{l}\right\|}_{2}^{2}}u_{l}=t_{j}^{i}-\frac{t_{j}^{i^{T}}u^{i}}{{\left\|u^{i}\right\|}_{2}^{2}}u^{i} \end{array},$$ (5)
  where, $r^{i}$ represents the residual vector of the i’th iteration, $t_{j}^{0}=a_{j}$, $a_{j}$ is the j’th column of A, $u^{i}$ represents the orthogonal basis vector of the i’th iteration.
2. Calculate sparsity $K^{i}$ and parameter $L^{i}$: conventional OLS methods need to preset the sparsity coefficient K empirically, whereas sparsity may not be available in many practical applications, which greatly limits the practicality of FMT reconstruction. Here, based on the initial sparsity $K^{0}$, we adopt the sparsity adaptive strategy. A nonlinear function was utilized to adjust the adaptive step size. It can be divided into two parts: (a) fast estimation of large step size in the initial stage, and (b) the completion stage is gradually approached in small steps. It is expressed as follows:
  $$K^{i}=K^{i-1}+\lceil K^{0}\cdot {\left(\frac{1}{i+1}\right)}^{2}\rceil ,$$ (6)
  $$L^{i}=L^{i-1}-\lceil L^{0}\cdot {\left(\frac{1}{i+1}\right)}^{2}\rceil ,$$ (7)
  where I is the iteration number, $L^{i}$ is the number of columns added to the support set in i’th iteration, $L^{0}$ is the initial parameter. ⌈·⌉ means round up. In initial stage, the step size takes a suitable initial value, and each time $K^{i}$ and $L^{i}$ are changed by Eqs. (6) and (7). The advantage of step change of nonlinear function is that the step change in the initial stage is large, and the step change in the completion stage is small, as shown in Fig. 1(a). This means the proposed method can approach the real sparsity more quickly. According to Eqs. (6) and (7), the sparsity $K^{i}$ increases, the parameter $L^{i}$ decreases.
3. Update the support set: considering that tumor always grows in a certain region, which when compared to the whole domain is sparse enough. To utilize the local spatial structure information sufficiently, we proposed a novel neighbor strategy based on the finite element theory. The neighbor set is constructed according to the tetrahedral element structure, as shown in Fig. 1(b). Suppose $S^{i}$ represents the support set generated from the i’th iteration. Let any node $G_{k}\in S^{i}$, then the nodes with tetrahedral edge connection relationship with node $G_{k}$ are the elements of the neighbor set. According to this rule, finding the neighborhood of all nodes in $S^{i}$ and uniting all neighborhood sets to form the final neighbor set $E^{i}$. The neighbor operator process is described by N(·), namely $E^{i}=N(S^{i})$. Selecting $L^{i+1}$ nodes from $E^{i}$ according to Eq. (3) and adding them into the support set $S^{i}$ to form a new support set $S^{i+1}$. That is, $S^{i+1}=L^{i+1}(E^{i})\cup S^{i}$, where $L^{i+1}(E^{i})$ indicates selecting $L^{i+1}$ nodes from $E^{i}$.
4. Update the residual: setting the $r^{i}$ as the residual vector represents the i’th iteration, where the residual vector required for the next iteration was formed as
  $$u^{i+1}≜q_{j},\;r^{i+1}=r^{i}-u^{i+1}.$$ (8)
5. Terminate the iteration: circulating the Steps (2) to (4), in each cycle i=i+1. The algorithm terminates when the halting condition is satisfied. The error reaching an acceptable range $r^{i}<\varepsilon$, the first $K^{i}$ columns of the final support set are solution sets.

**Algorithm 1:** Neighbor-based adaptive sparsity orthogonal least squares method.

Input: System matrix A, Surface photon distribution $\Phi^{m}$,
threshold ε, initial sparsity $K^{0}$, initial parameter $L^{0}$
Output: recovered support $S^{i}$, estimated signal x^
Initialize: support set $S^{0}=Ø$, iteration number i=1,
residual vector $r^{i}=\Phi^{m}$, $t_{j}^{i}=a_{j}$, $q_{j}=\frac{a_{j}^{T}r^{i}}{a_{j}^{T}t_{j}^{i}}t_{j}^{i}$, $E^{1}=N(S^{1})$
$S^{1}$ was generated from choosing $L^{0}$ columns by Eq. (3)
While: ${\left\|r^{i}\right\|}_{2}\geq \varepsilon$ do
1. Calculate sparsity $K^{i}$ and parameter $L^{i}$ by Eqs. (6) and (7);
2. Select $L^{i+1}$ largest terms from $E^{i}$ according to Eq. (3) into $S^{i}$, utilize neighbor operator N(·) to obtain neighbor set $E^{i}$;
3. i=i+1;
4. $S^{i+1}=L^{i+1}(E^{i})\cup S^{i}$, $L^{i+1}(E^{i})$ indicates to select $L^{i+1}$ nodes from $E^{i}$;
5. Perform (8) $L^{i}$ times to update ${\left\{U_{z}\right\}}_{z=1}^{L^{i}}$ and $r^{i}$;
6. $t_{j}^{i+1}=t_{j}^{i}-\frac{t_{j}^{i^{T}}u^{i}}{{\left\|u^{i}\right\|}_{2}^{2}}u^{i}$;
end while
7. the solution x^=Ki(Si)

## 3 Experiments and Results

In this section, experiments were carried out to evaluate the performance of NASOLS, including numerical simulation experiments with single target and double targets, phantom experiments with double targets, and small animal implanted experiments. The sparsity levels of ASOLS, OLS, CoSaMP, and gOMP are set to 4, 10, 8, and 6. The error in halting condition for different methods is set to 1e−8. The experiment codes were written in MATLAB and all these processes were executed on a desktop computer with 3.20 GHz Intel Processor i7-8700 CPU and 16 GB RAM. To quantify the reconstruction performance, location error (LE), normalized root-mean-square error (NRMSE), and contrast-to-noise ratio (CNR) were adopted in this study.^31^^,^^32^ The detailed formula descriptions can be found in the literature.^32^ In general, a high-quality reconstructed image possesses LE, NRMSE, close to 0 and a high CNR value.

**Fig. 2:**
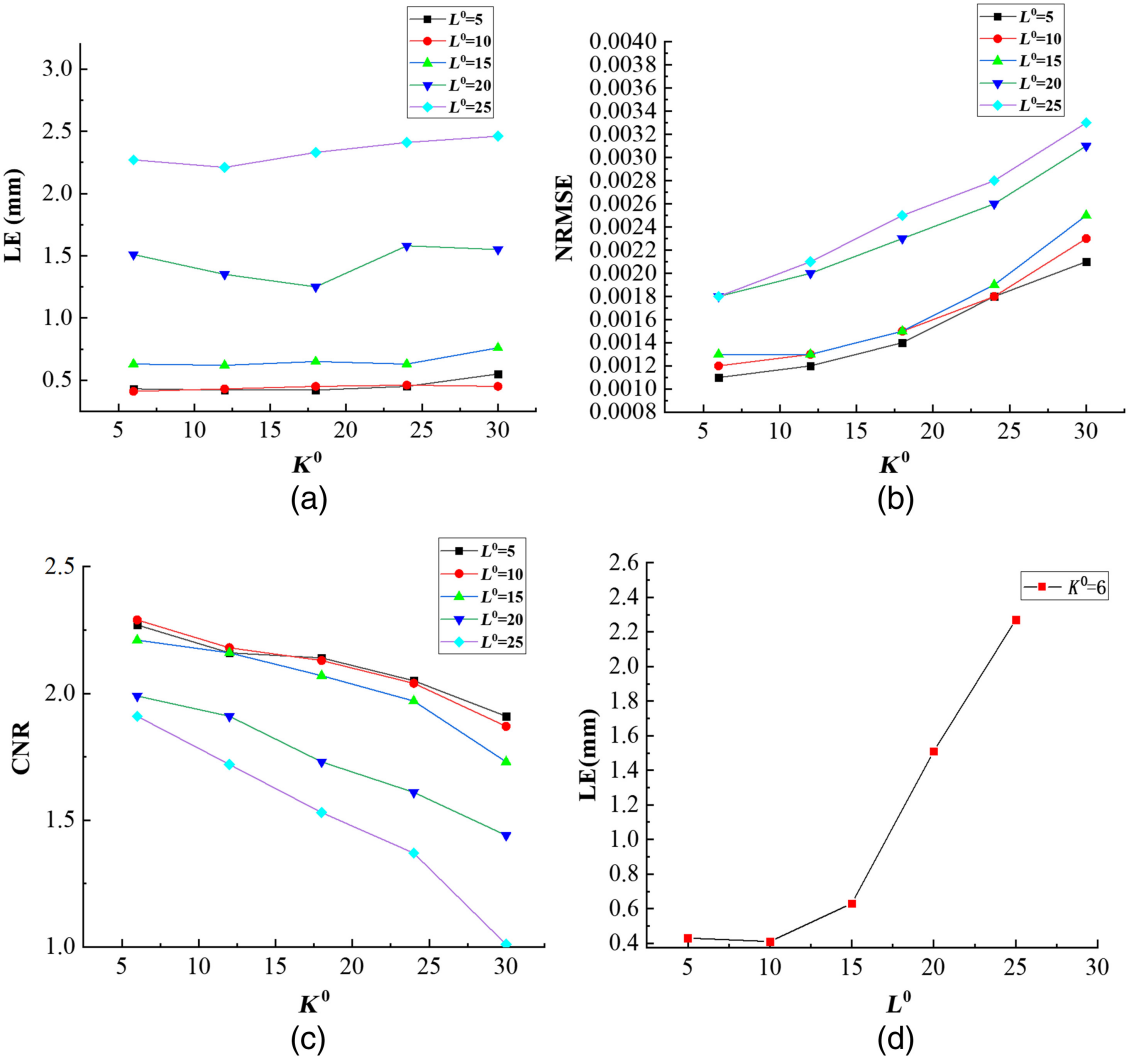
Quantitative comparison of parameter $L^{0}$ and $K^{0}$ test. (a)–(c) LE, NRMSE, and CNR for different $K^{0}$ and $L^{0}$. (d) Fixed $K^{0}=6$, the LE for different $L^{0}$.

As shown in Fig. 2, the initial value of $L^{0}$ have affected the recovered results. The initial value of $K^{0}$ and $L^{0}$ was determined based on the results of NASOLS. We set $K^{0}$ increased by 6 every time from 6 to 30, and $L^{0}$ increased by 5 every time from 5 to 25. Figure 2 shows the quantitative reconstruction results with different $L^{0}$ and $K^{0}$. It can be seen that when $L^{0}=10$, $K^{0}=6$, NASOLS achieved small LE, small NRMSE and large CNR. So the initial values of $K^{0}$ and $L^{0}$ in NASOLS are set to 6 and 10.

### 3.1 Numerical Experiments

**Fig. 3:**
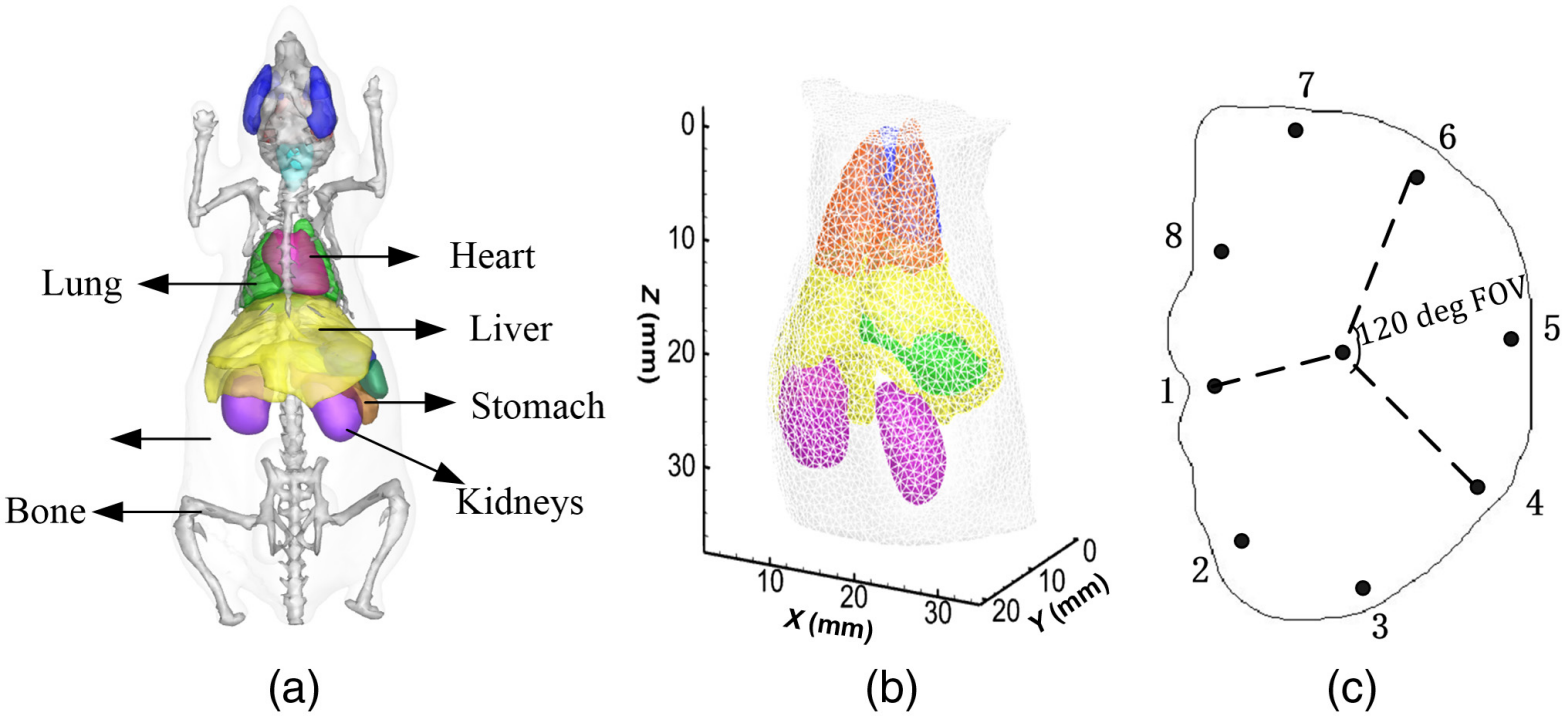
(a) 3D digital mouse model, (b) reconstruction mesh for the inverse problem, and (c) plane of excitation sources. The black points are the location of the isotropic point sources. For each excitation source, fluorescence is detected at the opposite side with a 120 deg FOV.

A 3D digital mouse model was employed,^31^ as shown in Fig. 3(a). The torso section of the mouse, including heart, lung, liver, stomach, kidneys, and muscle, was the investigated region. The optical parameters of the main organs were the same as used in literature.^13^^,^^33^^,^^34^ Figure 3(b) shows the reconstruction mesh of the torso section for the inverse problem. It contains 5890 nodes and 29,308 tetrahedral elements. Here, the fluorescent target was excited by eight point sources at different positions in sequence as shown in Fig. 3(c). The black dots represent positions of the excitation point sources, which were modeled as isotropic point sources located one mean free path of photon transport beneath the surface. For each excitation source, the surface data on the opposite side with a 120 deg field of view (FOV) were measurable.

#### 3.1.1 Single target reconstruction

**Fig. 4:**
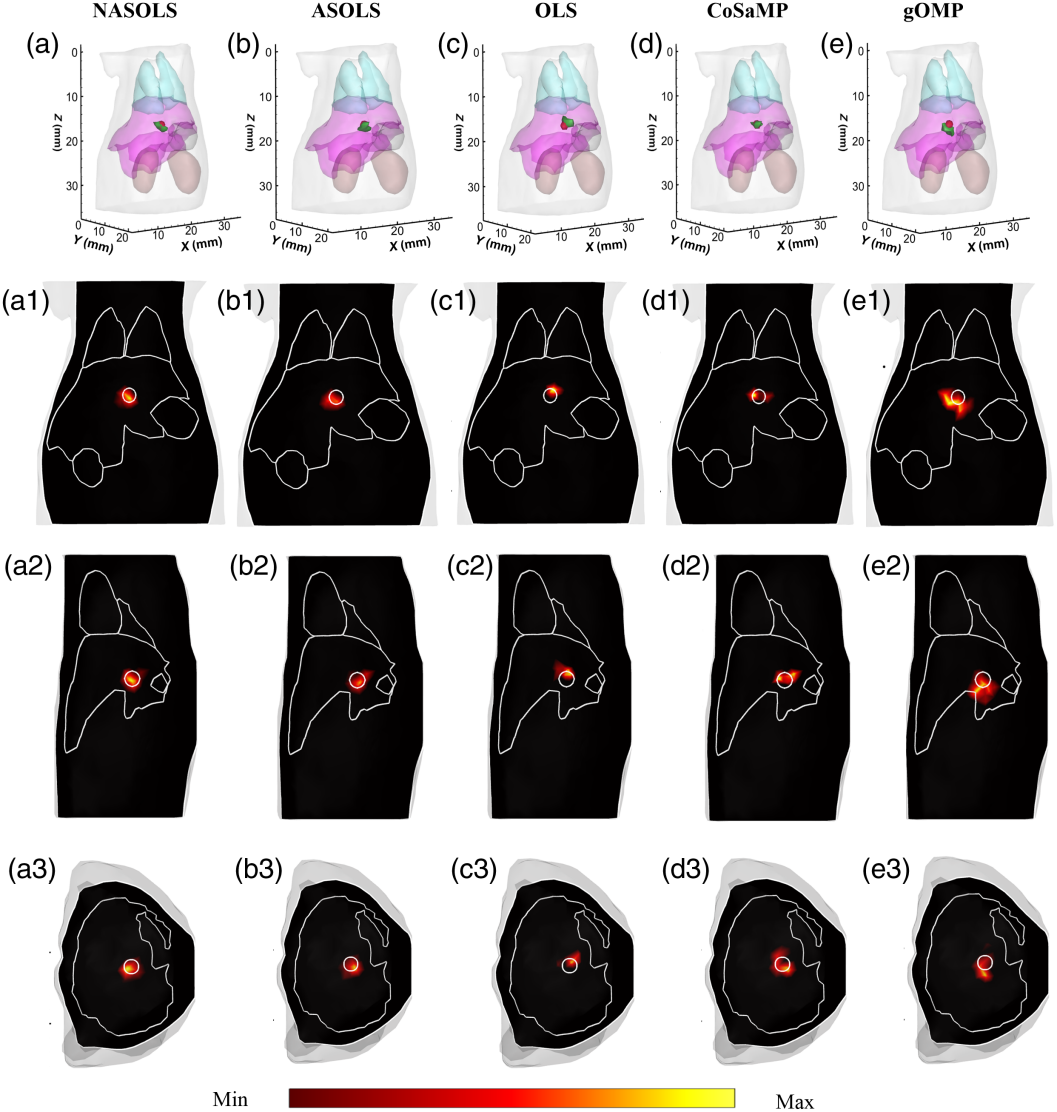
(a)–(e) Recovered results by 3D views. The red spheres represent the actual fluorescent target and the green areas denote the reconstructed one. (a1)–(e1) Sagittal slices, (a2)–(e2) coronal slices, and (a3)–(e3) transverse slices. The white circles denote the real target.

Figure 4 shows the reconstructed results with eight projections measurements. In the single target experiment, a sphere with radius of 1 mm was placed in the liver with center at (18.0, 12.0, 16.4 mm). The fluorescent yield of the target was set to be $0.05\;{\mathrm{mm}}^{-1}$. In the forward problem, the digital mouse model is a mesh with 24,041 nodes and 1,27,248 tetrahedral elements. The first row is the 3D views of the FMT images reconstructed using the NASOLS, ASOLS, OLS, CoSaMP, and gOMP, respectively. The red sphere is the real target, and the green area is the reconstructed target, respectively. The second column is the coronal slices, the third column is the sagittal slices, and the fourth column is the transverse slices. The white circle denotes the actual position of the target. The corresponding quantitative indicators are given in Table 1. It is obvious that the proposed algorithm achieved the smallest LE and NRMSE and largest CNR. It provided better results than the other four approaches, which indicated that our method has the ability for the single target reconstruction.

**Table 1:** Quantitative results of single target reconstruction experiment.

Method	Center (mm)	LE (mm)	NRMSE	CNR
NASOLS	(17.81,12.06,16.02)	**0.41**	**0.0012**	**2.29**
ASOLS	(17.51,12.16,16.65)	0.57	**0.0012**	1.61
OLS	(18.32,12.18,17.03)	0.73	0.0016	1.58
CoSaMP	(17.59,12.12,16.92)	0.66	0.0014	0.84
gOMP	(17.09,12.23,16.54)	0.89	0.0014	1.52

Note: The best results are in bold.

**Fig. 5:**
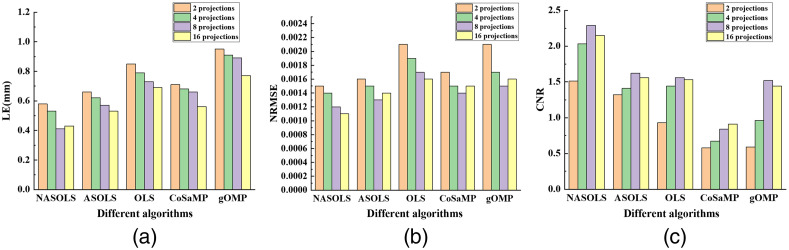
The quantitative analysis of single target with different projection numbers by NASOLS, ASOLS, OLS, CoSaMP, and gOMP. (a)–(c) LE, NRMSE, and CNR of five methods.

As shown in Fig. 5, different projection numbers (2, 4, 8, 16) simulations have been conducted to show the relationship between the number of projections and the reconstruction accuracies of different algorithms. In the experiments, take projection number of 16 as example, NASOLS provides the smallest LE, NRMSE and the largest CNR compared to the other four methods. With the increasing of projection number, the recovered results became more accurate for most cases. However, NASOLS provided largest CNR with 8 projections, not 16 projections, so did ASOLS, OLS, and gOMP. The reason may be that the redundant information appeared with the increasing of projection numbers, which in turn affected the reconstruction accuracy.

**Fig. 6:**
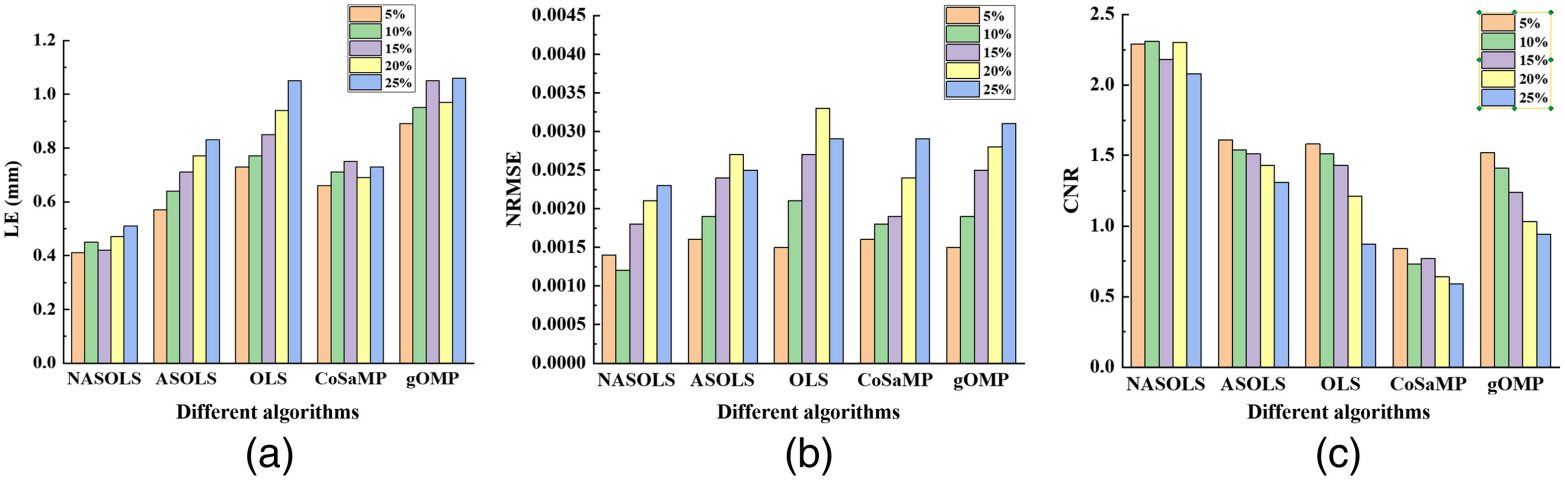
Quantitative results of single target with different levels of noise (5%, 10%, 15%, 20%, and 25% Gaussian noise). (a)–(c) LE, NRMSE, and CNR of five methods.

Experiments with different levels of noise (5%, 10%, 15%, 20%, and 25% Gaussian noise) of the measurements were carried out to study the effect of noise on the reconstruction results. It was clear that the recovered results became worse with the increasing of the noise levels, as shown in Fig. 6. However, NASOLS performed best of five methods according to LE, NRMSE, CNR.

#### 3.1.2 Double-target reconstruction

In this section, double targets with different edge to edge distance (EED) were designed to further show the performance of the algorithm. Two spheres with radius of 1 mm were placed in the center of (16.0, 14.5, 14.0 mm) and (20.0, 14.5, 14.0 mm), respectively.

**Fig. 7:**
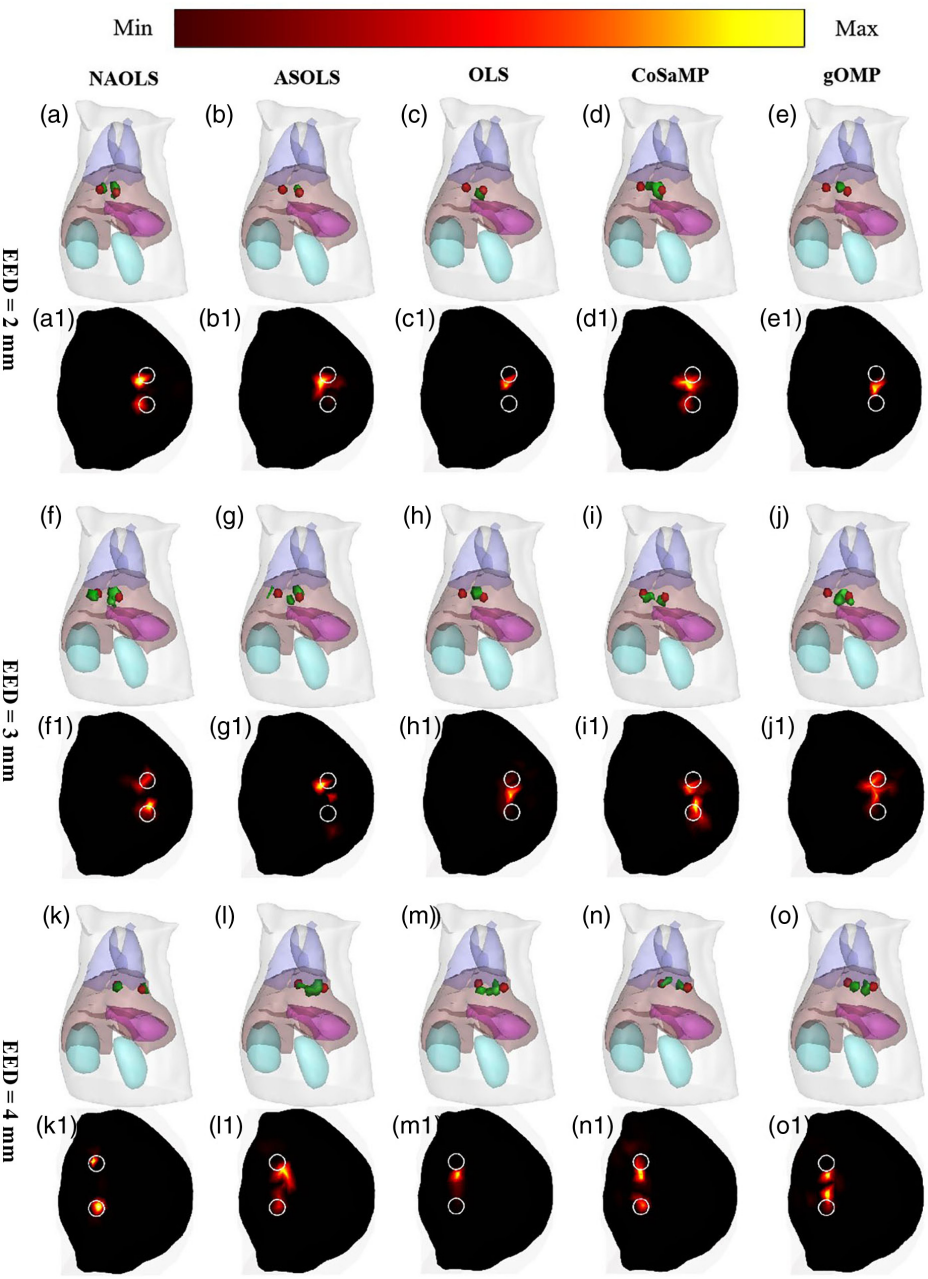
Double-target recovered results with different EEDs. (a)–(e) 3D view results when the EED is 2 mm by NASOLS, ASOLS, OLS, CoSaMP, and gOMP. (a1)–(e1) Transverse slices at z=14.0  mm. (f)–(j) 3D view results when the EED is 3 mm by the five methods. (f1)–(j1) Transverse slices at z=14.0  mm. (k)–(o) 3D view results when the EED is 4 mm. (k1)–(o1) Transverse slices at z=14.0  mm. The white circles represent the real targets.

The EED of the two spheres is 2 mm, and the forward mesh had 24,275 nodes and 1,28,635 tetrahedral elements. When the EED was set to be 3 mm, the center of two targets were (15.0, 14.5, 14.0 mm) and (20.0, 14.5, 14.0 mm). Its forward mesh of digital mouse concluded 19,306 nodes and 1,01,362 tetrahedral elements. When the EED was set to be 4 mm, the center of two targets were (16.0, 14.5, 14.0 mm) and (22.0, 14.5, 14.0 mm). In total, 19,339 nodes and 1,01,562 tetrahedral elements were included in the forward mesh. The difficulty of reconstruction increases with the decrease of EED, so the double-target experiments with different EEDs could reflect the spatial resolution. Figures 7(a)–7(e) show the reconstructed results when the EED was 2 mm. When the EED was 2 mm, ASOLS, gOMP, and OLS cannot distinguish two targets. Both of NASOLS and CoSaMP had the ability to distinguish two targets. But, there were a lot of artifacts by CoSaMP reconstruction. Figures 7(f)–7(j) show the recovered results when the EED was 3 mm. gOMP and OLS could only reconstruct one target, which was close to the middle of the two targets. ASOLS could reconstruction one target accuracy. NASOLS and CoSaMP could reconstruct two targets. However, the artifact of one target reconstructed by CoSaMP is large, the location error of two targets by CoSaMP is larger than NASOLS. Figures 7(k)–7(o) show the reconstruction results when the EED was 4mm. OLS could only recover one target. NASOLS could recover the two targets very well, which was better than the other four algorithms. From the quantitative results in Table 2, NASOLS had the smallest LE, the smallest NRMSE, and the largest CNR when EED was 2, 3, or 4 mm.

**Table 2:** Quantitative results of different EED experiments.

EED (mm)	Methods	Center (mm)	LE1 (mm)	LE2 (mm)	NRMSE	CNR
2	NASOLS	(19.10,13.57,14.09)	**1.29**	**1.02**	**0.0035**	**1.84**
		(16.20,13.50,13.82)				
	ASOLS	(18.79,13.55,14.15)	1.54	2.13	0.0041	1.33
		(17.76,13.29,13.91)				
	OLS	(18.92,13.87,14.89)	1.57	3.51	0.0042	1.32
		(13.00,15.52,12.50)				
	CoSaMP	(18.43,14.02,14.11)	1.64	1.28	0.0064	0.82
		(17.01,13.83,13.56)				
	gOMP	(18.39,14.34,15.53)	2.20	2.01	0.0049	0.63
		(17.83,13.67,13.65)				
3	NASOLS	(19.60,14.22,14.85)	**1.00**	**0.88**	**0.0027**	**2.28**
		(15.58,13.95,14.46)				
	ASOLS	(19.37,13.59,13.82)	1.12	1.65	0.0033	1.89
		(16.35,13.59,13.74)				
	OLS	(18.60,13.79,13.88)	1.51	2.89	0.0035	1.88
		(17.85,14.14,13.68)				
	CoSaMP	(19.22,13.39,13.59)	1.43	1.01	0.0053	1.01
		(15.07,14.55,15.06)				
	gOMP	(18.22,14.08,13.77)	1.81	1.82	0.0031	0.95
		(16.78,14.70,13.71)				
4	NASOLS	(22.13,7.10,14.10)	**0.44**	**0.70**	**0.0011**	**2.83**
		(16.66,7.77,14.02)				
	ASOLS	(20.89,8.02,13.95)	1.22	0.97	0.0023	2.39
		(16.93,7.76,14.08)				
	OLS	(20.07,7.78,14.21)	1.95	1.46	0.0025	2.29
		(17.07,8.03,14.83)				
	CoSaMP	(20.73,7.46,13.92)	1.26	0.89	0.0022	1.62
		(16.70,7.27,13.49)				
	gOMP	(20.51,7.52,14.61)	1.45	1.47	0.0027	1.55
		(17.19,6.98,13.32)				

Note: The best results are in bold.

**Fig. 8:**
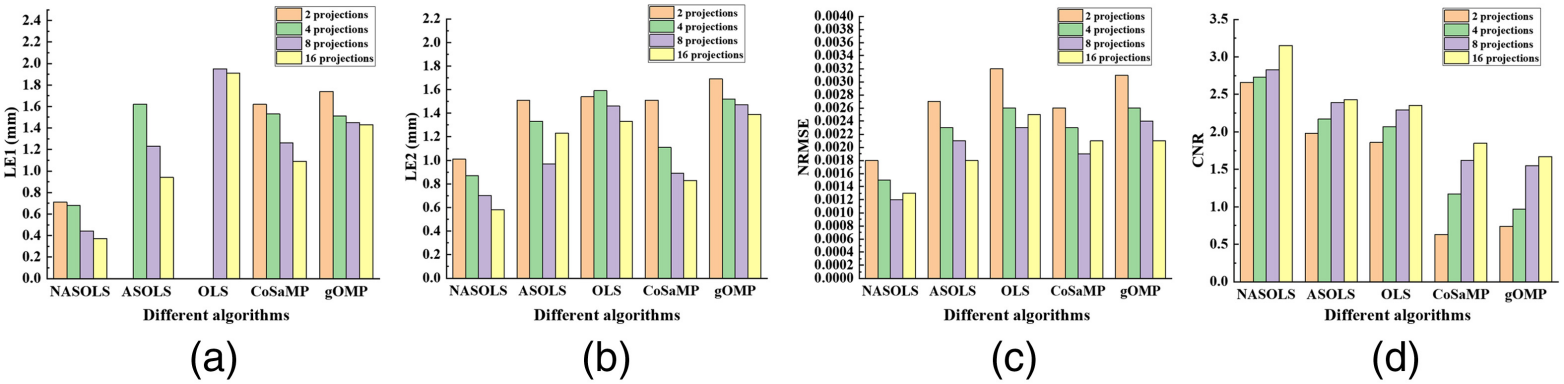
The quantitative analysis of double targets with different projection numbers by NASOLS, ASOLS, OLS, CoSaMP, and gOMP. (a)–(d) LE1 (Target 1), LE2 (Target 2), NRMSE, and CNR of five methods.

Double targets (EDD at 4 mm) with different projection numbers (2, 4, 8, 16) were also carried out and the recovered results are shown in Fig. 8. Take projection number of 16 as an example, NASOLS has also performed best according to LE, NRMSE, and CNR compared to the other four methods. In addition, we have found that when the projection number is too small, ASOLS and OLS cannot distinguish two targets. For example, OLS could only recover one target with projection numbers 2 and 4, so did ASOLS with 2 projections. Then, it can be seen that there is no result for these cases, as shown in Fig. 8(a).

**Fig. 9:**
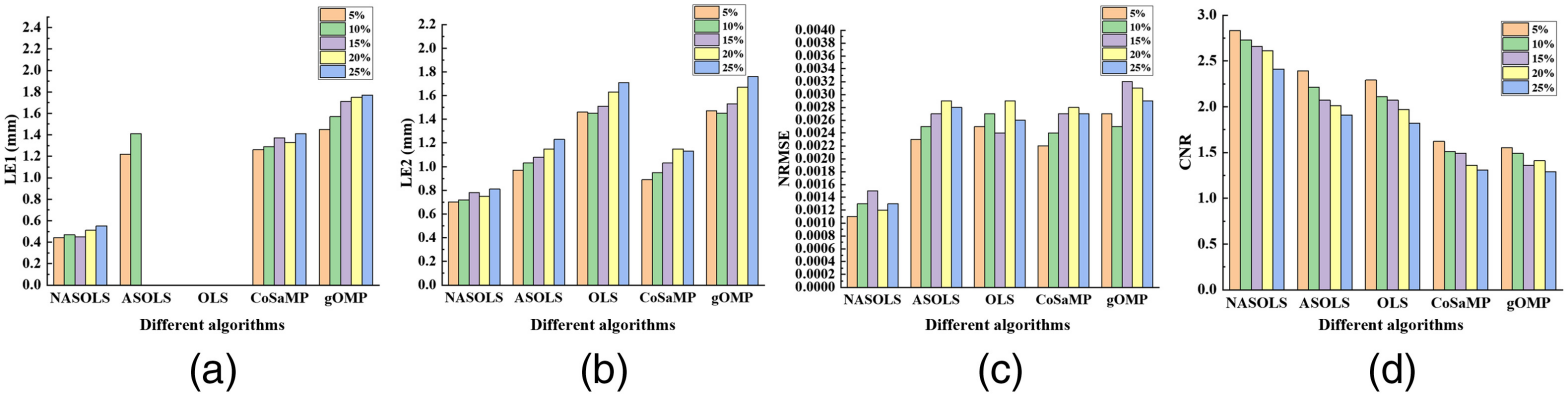
The quantitative analysis of double targets with different levels of noise by NASOLS, ASOLS, OLS, CoSaMP, and gOMP. (a)–(d) LE1 (Target 1), LE2 (Target 2), NRMSE, and CNR of five methods.

Double-target (EDD at 4 mm) experiments with different levels of noise (5%, 10%, 15%, 20%, and 25% Gaussian noise) of the measurements have also been conducted. From Fig. 9(a), it is clear that OLS cannot distinguish two targets if there was noise on the data. It indicated that OLS is sensitive to noise. When noise level is 15%, 20%, and 25%, ASOLS cannot distinguish either. Anyway, NASOLS provided best results according to LE, NRMSE, and CNR.

### 3.2 Real Experiments

**Fig. 10:**
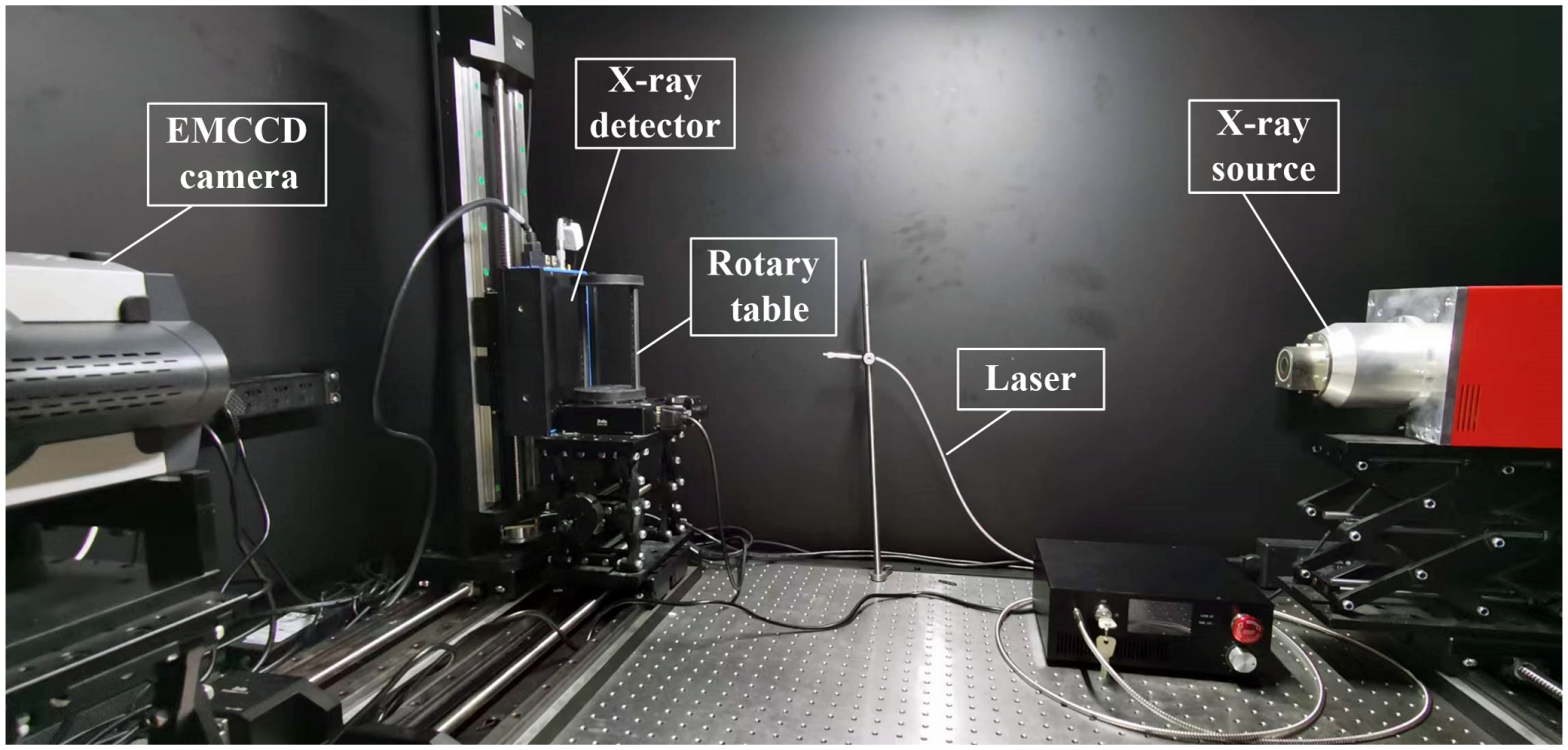
FMT/micro-CT dual modality imaging system.

In this section, physical phantom and small animal experiments were conducted to further evaluate the algorithm. First, the real fluorescence data was collected by the FMT/micro-computed tomography (CT) dual modality imaging system, as shown in Fig. 10. It is a full-angle, non-contact imaging system. This system could obtain fluorescence signals and structural information simultaneously. The excitation light with 670 nm wavelength was provided by a continuous wave laser source (CrystaLaser, Reno, Nevada, and Model No. CL671-050-O). The rotational stage could rotate by the computer. And a highly sensitive charge-coupled device (CCD) camera (Princeton Instruments PIXIS 2048B, Roper Scientific, Trenton, New Jersey, United States), which was cooled to −80°C to reduce the effects of thermal noise, was used to collect the emitted light. Micro-CT system includes an x-ray detector (1512N-C90-HRCC, Dexela, United States) and an x-ray generator (L9181-02 MT2195, Hamamatsu Photonics, China). The control module has a 360-deg motorized turntable (RAK100, Zolix, China) and a controller (Zolix Instruments Co., Beijing, China). The x-ray source and x-ray detector are placed on the same line. In addition, The CCD was perpendicular with this line. Different projections of fluorescence images could be obtained by rotating the stage. In general, optical images were collected first, and then the CT data were followed by the micro-CT systems. Main organs could be segmented from the CT data, and a heterogeneous mouse model could be obtained. The optical parameter of main organs were the same as used in literature.^13^ A mesh for the inverse problem was provided by discretizing heterogeneous mouse model. The fluorescence image was mapped to the mesh to obtain the measurement data.^35^

#### 3.2.1 Physical phantom experiment

**Fig. 11:**
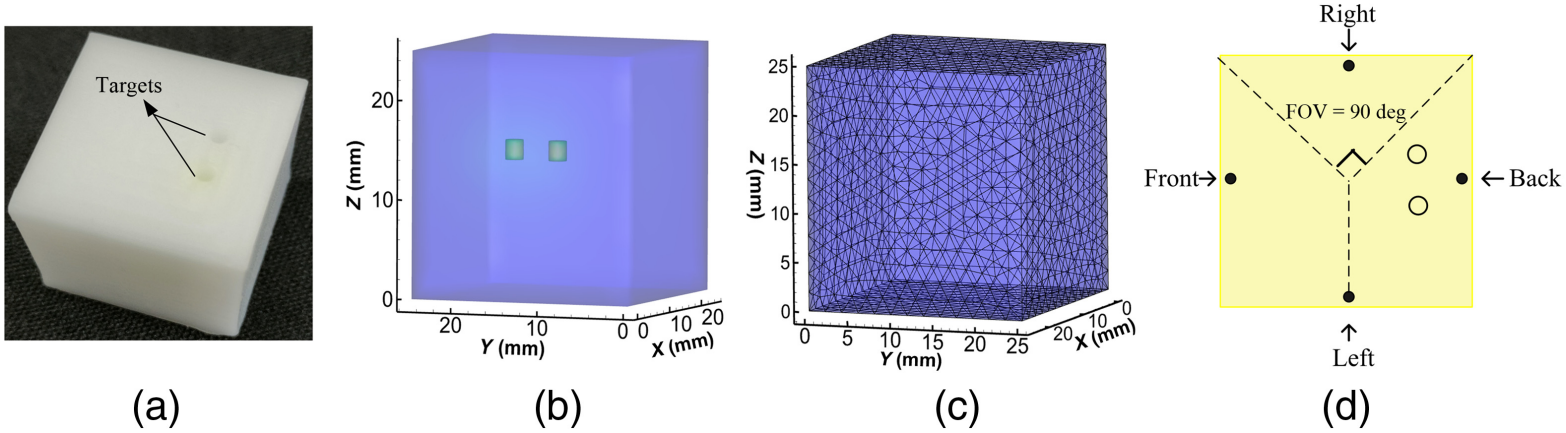
(a) Physical phantom; (b) geometric structure of the cubic phantom with double targets (2 mm in diameter and 2 mm in height); (c) reconstruction mesh for the inverse problem; (d) x-y view on the z=15.0  mm plane, where the black dots represent the excitation point source positions. Four degrees show the direction of the CCD camera during data acquisition.

The phantom, a cube with a side length of 25 mm, was made of polyformaldehyde. Its optical parameters for both excitation and emission wavelengths are the same as illustrated in Ref. 36, which were determined by diffuse optical tomography. Two small holes with 1 mm radius were drilled to emplace the Cy5.5 solution. The Cy5.5 solution was injected into the holes, which became two cylindrical targets with a height of 2.0 mm. Their centers were (5.0, 10.0, 15.0 mm) and (5.0, 15.0, 15.0 mm) with EED of 3 mm, as shown in Figs. 11(a) and 11(b). The mesh for the inverse reconstruction was discretized into 6031 nodes and 32,430 tetrahedral elements. Here, four projections were acquired by rotating the phantom with an angular increment of 90 deg. The fluorescent targets were excited by point sources from four different positions at the z=15.0  mm plane and CCD acquired data at four different views, as shown in Fig. 11(d).

**Fig. 12:**
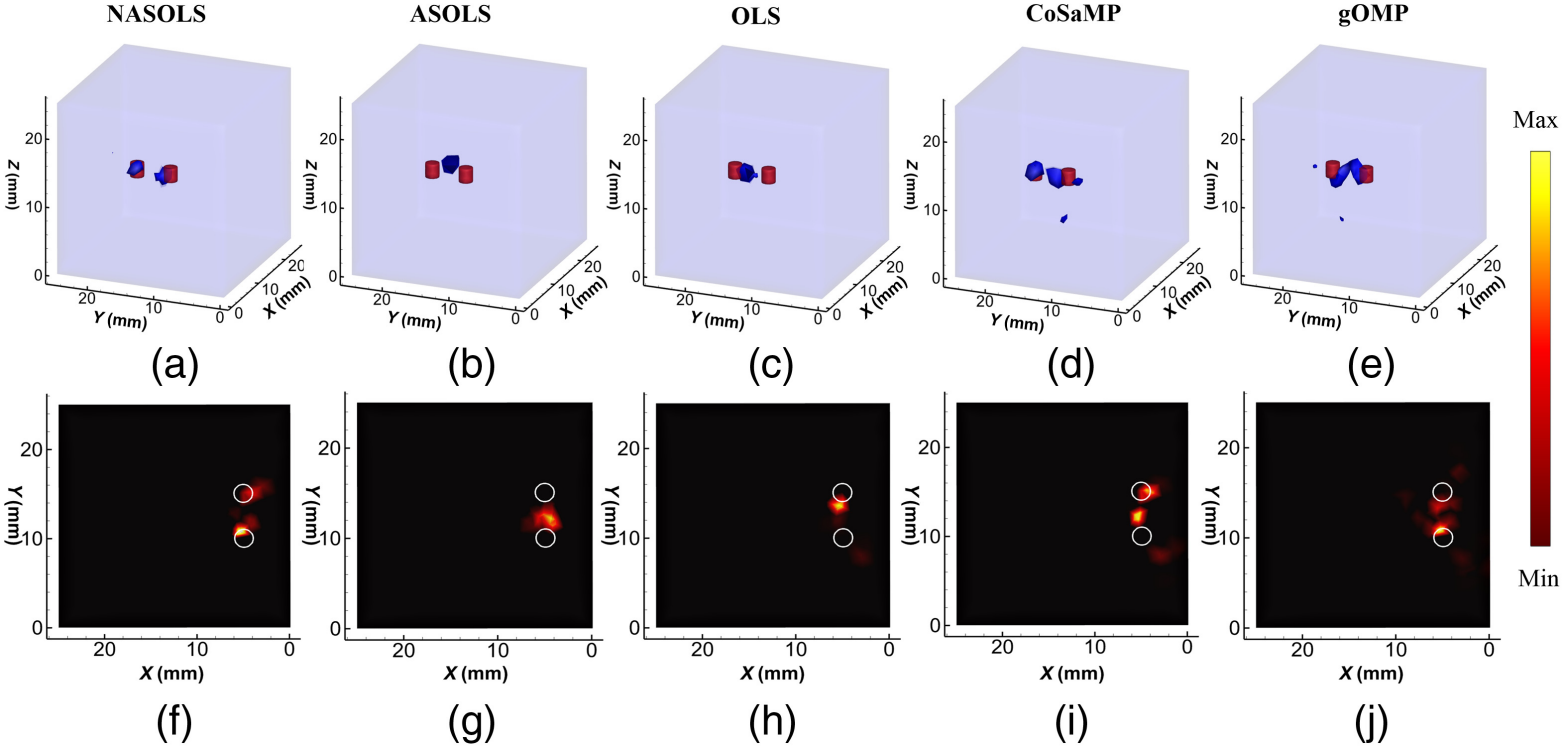
The phantom experiments results. (a)–(e) 3D view of results using NASOLS, ASOLS, OLS, CoSaMP, and gOMP, respectively. (f)–(j) Transverse views at Z=15  mm slices.

Figure 12 shows reconstruction results with 3D and transverse views. It is clear that the proposed method could distinguish two targets while ASOLS, OLS, and gOMP could not do from the 3D views, shown in Figs. 12(a)–12(e). For CoSaMP method, one of the reconstructed targets was in the middle of two actual targets, and the artifacts appeared, which would mislead researchers. The transverse views in Figs. 12(f)–12(j) had also given the similar conclusion. From Table 3, the LE by NASOLS is the smallest, NRMSE is the smallest, and CNR is the largest. This means NASOLS provided the best results of the five methods.

**Table 3:** Quantitative results of double-target reconstruction in physical phantom experiment.

Methods	Center (mm)	LE1 (mm)	LE2 (mm)	NRMSE	CNR
NASOLS	(4.48,14.92,15.44)	**0.68**	**0.80**	**0.0082**	**2.82**
	(5.36,10.65,14.70)				
ASOLS	(4.38,11.62,14.53)	1.81	—	0.0092	2.42
	—				
OLS	(5.44,13.50,14.60)	1.61	—	0.0099	2.42
	—				
CoSaMP	(4.48,14.92,15.44)	**0.68**	2.37	0.0087	1.56
	(5.62,12.21,14.43)				
gOMP	(4.37,12.32,14.14)	2.88	0.96	0.011	2.31
	(5.09,10.95,15.06)				

Note: The best results are in bold.

**Fig. 13:**
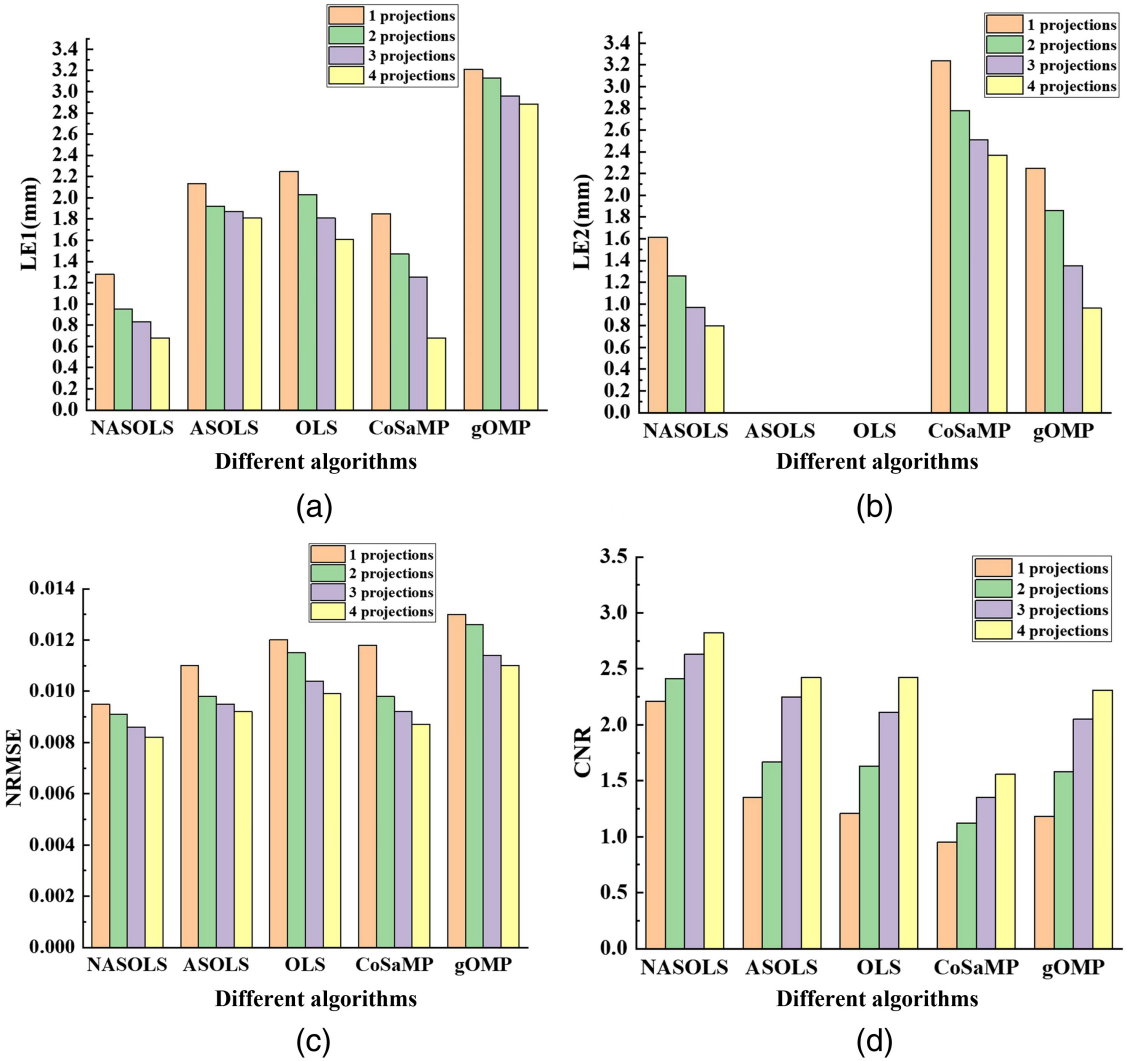
The quantitative analysis of phantom experiments (two targets) with different projections (1, 2, 3, 4) for five methods: (a) and (b) LEs, (c) NRMSE, and (d) CNR.

The phantom experiments with different projections (1, 2, 3, 4) were carried out too. Figure 13 showed the recovered results for five methods. With the increasing of projection numbers, the reconstruction results were improved according to the evaluation index for five methods. It is clear that the quantitative indicators of the results with four projections were better than those of the results with one projection. And NASOLS provided the best results. In addition, ASOLS and OLS algorithms can only reconstruct one target, then there is no result in Fig. 13(b).

#### 3.2.2 *In-vivo* experiments

To further evaluate the performance of the proposed algorithm, implanted *in-vivo* experiments were performed on two small mice. All animal studies were performed in accordance with the Fourth Military Medical University Guide for the Care and Use of Laboratory Animals formulated by the National Society for Medical Research. To relieve the pain of the mouse, the experiment was conducted under isoflurane gas anesthesia. In addition, Cy5.5 solution with concentration of 4000 nM was injected into the glass tube with a diameter of 2.1 mm and a height of 2.8 mm. The glass tube was implanted into the adult mouse (excitation spectrum at 671 nm, emission spectrum at 710 nm). First, we collected the fluorescence images and then the CT data. After segmentation, we can obtain the main organs of mouse, including heart, lung, liver, kidney, and muscle, etc.

**Fig. 14:**
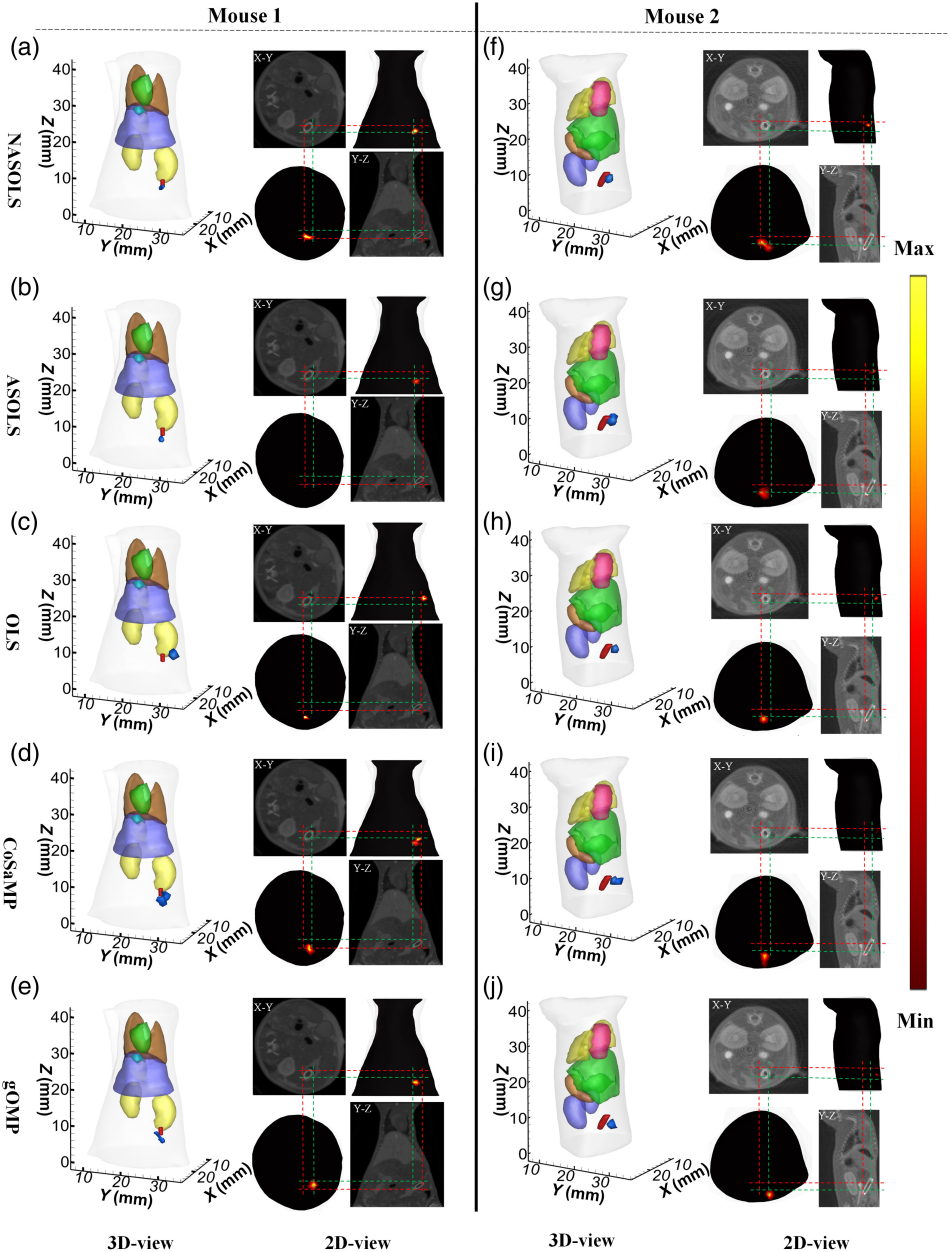
*In-vivo* experiment results on small animals. (a)–(e) Recovered results for Mouse 1 by NASOLS, ASOLS, OLS, CoSaMP, and gOMP, respectively. (g)–(j) Reconstruction results for Mouse 2 by NASOLS, ASOLS, OLS, CoSaMP and gOMP, respectively. In the 3D views, the red region represents the real target and the blue region is the recovered results.

Figure 14 shows the recovered results on 3D views and 2D views combined with CT for two mice. For two mice, the real center locations of the targets are at (19.8, 27.1, 8.1 mm) and (14.6, 19.4, 7.1 mm), respectively. The inverse mesh of Mouse 1 has 3878 nodes and 18,866 tetrahedral elements while the mesh of Mouse 2 has 9466 nodes and 47,631 tetrahedral elements. Table 4 shows the quantitative results for two mice by NASOLS, ASOLS, OLS, CoSaMP and gOMP, respectively. In the 3D views, the real target and reconstructed target were depicted with red and blue regions respectively. It is clear that OLS and gOMP algorithms has provided a deviated result with great error, whereas NSAOLS can locate the target center more accurately according to the 3D views and transverse views for both two mice. Similar conclusions can also be drawn from LE, NRMSE, CNR of Table 4. It can be concluded that NASOLS shows good advantages compared with the other four methods.

**Table 4:** Quantitative results of *in-vivo* experiment.

	Methods	Center (mm)	LE (mm)	NRMSE	CNR
Mouse 1	NASOLS	(19.6,27.4,6.9)	**1.2**	**0.0013**	**0.49**
	ASOLS	(19.3,26.2,5.8)	2.4	0.0015	0.31
	OLS	(16.9,23.6,6.7)	4.7	0.0024	0.29
	CoSaMP	(19.6,27.1,5.9)	2.1	0.0017	0.19
	gOMP	(18.8,25.4,5.4)	3.3	0.0020	0.20
Mouse 2	NASOLS	(14.0,20.7,7.3)	**1.4**	**0.0010**	**1.02**
	ASOLS	(13.1,20.9,7.5)	2.1	0.0012	0.66
	OLS	(12.1,17.5,5.7)	3.4	0.0013	0.47
	CoSaMP	(13.5,21.4,7.4)	2.2	0.0010	0.87
	gOMP	(13.5,21.6,7.5)	2.7	0.0015	0.58

Note: The best results are in bold.

## 4 Discussion and Conclusion

In this paper, we proposed an adaptive sparsity with neighbor strategy orthogonal least-squares to solve $L_{0}$-norm regularization problem in FMT. An adaptive strategy was proposed to enhance the practicability of our algorithm. A nonlinear function was presented to adjust the sparsity step, so the estimated sparsity can quickly approach the real sparsity. Then, the nearest neighbor strategy based on a finite-element tetrahedral mesh structure was used to form the support set, which has enhanced the local connection characteristic. Actually, the optimal solution based on FEM was usually distributed around the maximum energy node. The nearest neighbor idea was taking advantage of this property, so the developed method could provide more accurate recovered results, especially for the double-target reconstruction. From the double-target simulation experiments, NASOLS could clearly distinguish two targets with EED at 2, 3, and 4 mm. In fact, FMT is heavily influenced by light scattering and it is difficult to achieve such resolutions in the real biological applications. Here, the double-target reconstructions were carried out in a completely ideal environment. The external noise, the error caused by the real experimental measurement and so on were not considered here. In the real experiments, all these external factors will also interfere with the reconstruction results and it is hard to achieve such resolutions. Different projection numbers in the single-target and double-target simulation. It demonstrated that the experimental results had become better with the increasing of projection numbers. However, the results became worse when the number of projections reached a certain number. For example, the recovered results with 16 projections were not better than those of eight projections in the simulations according to the evaluating indicators. The reason might be that the redundant information appeared with the increasing of projection numbers, which in turn affected the reconstruction accuracy. In addition, different levels of noise were added to the measurements and the results showed that as the levels of noise increased, the recovered results has become worse according to the evaluating indicators.

The accuracy of photon propagation model could be improved by combining anatomical information acquired from CT or magnetic resonance imaging into FMT reconstructions. In this work, the measurement data were detected by a FMT/micro-CT dual imaging system. The anatomical information were provided by micro-CT. Some researchers have introduced diffusion optical tomography (DOT) into FMT to provide optical parameters, which further improved the quality of FMT.^37^ However, the inverse problem of DOT is also severely ill-conditioned and ill-posed, and its reconstruction results are still susceptible to noise.

Apart from the traditional iteration-based regularization methods, deep learning has become one of the fastest-growing breakthrough technologies in recent years. There are generally two types of deep learning methods for FMT, namely end-to-end deep neural network and post processing methods. Traditional methods can provide the mathematical model from physical theory while deep learning is difficult to give a theoretical explanation for the FMT forward model. In addition, the design of network architecture and training schemes determine the performance of deep learning-based methods. Even so, deep learning based methods still has unprecedented advantages in FMT.

In summary, NASOLS algorithm improves the reconstruction performance of FMT from the experimental results. Therefore, this new method can promote the practical application of FMT in clinic. However, there are still some deficiencies. First, the selection of nearest neighbor nodes might be repeated in the reconstruction process, which resulted in time consuming of reconstruction process. Second, the NASOLS algorithm could improve the target location but its ability to reconstruct the shape of target was poor. As we all know, the shape reconstruction of FMT is very important and it is still a great challenge in practical applications. We will continue to work towards this shape reconstruction of target in the future.
